# Interpretations of legal criteria for involuntary psychiatric admission: a qualitative analysis

**DOI:** 10.1186/s12913-014-0500-x

**Published:** 2014-10-25

**Authors:** Eli Feiring, Kristian N Ugstad

**Affiliations:** Department of Health Management and Health Economics, University of Oslo, PO Box 1089, Blindern, Oslo, 0317 Norway

**Keywords:** Psychiatric care, Coercion, Paternalism, Qualitative, Legal norms, Professional ethics

## Abstract

**Background:**

The use of involuntary admission in psychiatry may be necessary to enable treatment and prevent harm, yet remains controversial. Mental health laws in high-income countries typically permit coercive treatment of persons with mental disorders to restore health or prevent future harm. Criteria intended to regulate practice leave scope for discretion. The values and beliefs of staff may become a determinating factor for decisions. Previous research has only to a limited degree addressed how legal criteria for involuntary psychiatric admission are interpreted by clinical decision-makers. We examined clinicians’ interpretations of criteria for involuntary admission under the Norwegian Mental Health Care Act. This act applies a status approach, whereby involuntary admission can be used at the presence of mental disorder and need for treatment or perceived risk to the patient or others. Further, best interest assessments carry a large justificatory burden and open for a range of extra-legislative factors to be considered.

**Methods:**

Deductive thematic analysis was used. Three ideal types of attitudes-to-coercion were developed, denoted *paternalistic, deliberative* and *interpretive*. Semi-structured, in-depth interviews with 10 Norwegian clinicians with experience from admissions to psychiatric care were carried out. Data was fit into the preconceived analytical frame. We hypothesised that the data would mirror the recent shift from paternalism towards a more human rights focused approach in modern mental health care.

**Results:**

The paternalistic perspective was, however, clearly expressed in the data. Involuntary admission was considered to be in the patient’s best interest, and patients suffering from serious mental disorder were assumed to lack decision-making capacity. In addition to assessment of need, outcome effectiveness and risk of harm, extra-legislative factors such as patients’ functioning, experience, resistance, networks, and follow-up options were told to influence decisions. Variation in how these multiple factors were taken into consideration was found. Some of the participants’ statements could be attributed to the deliberative perspective, most of which concerned participants’ beliefs about an ideal decision-making situation.

**Conclusions:**

Our data suggest how a deliberative-oriented ideal of reasoning about legal criteria for involuntary admission lapses into paternalism in clinical decision-making. Supplementary professional guidelines should be developed.

## Background

In the 1980-ies, a cultural shift towards more user-orientation took place in public policy formation in high-income countries. The users should no longer be treated as passive recipients of welfare, nor should service providers behave like altruistic paternalists defining what was in the best interest of the recipient [1]. Accordingly, the paternalistic clinician-patient relationship was challenged, and a more active role for the patient was called for. Discussions about the use of coercive measures in medicine were intense and new legislation that place greater weight on patient autonomy and human dignity emerged [2]. A human rights-orientation has since then become increasingly influential in the development of mental health care law.

Still, as Wealsh and Deahl put it: “The power to detain and to treat a person against their will forms the backbone of the psychiatric profession” [3]. Coercive measures are used extensively in modern mental health care. This practice is, however, controversial. It is a serious deprivation of individual liberty and autonomy and may be experienced as gravely humiliating, even violent [4,5]. It is also shown to affect marginalised persons more than others [6].

Involuntary admission and treatment have traditionally been justified as measures to prevent suffering and to give appropriate care, or offer protection from harm to oneself and to others. These arguments often rest upon four empirical premises. First, the person involved is thought incapable of making rational decisions about treatment. Second, the person involved will later be grateful. Third, coercive measures are effective. Four, risk assessment of dangerousness to oneself or others is reliable and valid.

Recent studies have demonstrated, however, that these premises are without conclusive research-based support. There is now evident that mentally ill patients may have decision-making capacity [7-9], although evidence also suggests that clinicians overestimate their patients’ competence [9]. Further, there is little empirical knowledge about the effects of involuntary care and the few studies available have shown contradictory results [2,4,6,10]. Also, it is yet not known whether use of structured risk assessment instruments improves practice [11]. The implication is that the beneficiary effects of coercion are harder to defend. Both professionally and politically, discussions about the use and possible reduction are called for [2,3].

Prior research has shown that the use of coercion varies both within and between jurisdictions [12-14]. Differences and changes in health care organisation and financing, legislation, socio-demographic characteristics, diagnostic patterns and patient characteristics play important roles in explanations of such variations, but findings are inconclusive [6,15-17] Differences within countries also suggest that local treatment cultures and staff attitudes towards coercive measures may contribute to variations [14,18]. In a multilevel analysis of attitudes to coercion, some differences among wards were found, but most variance could be attributed to individual staff level factors within wards [19].

A few quantitative studies of attitudes towards different types of coercion have been carried out. In a national U.S. survey of psychiatrists’ views about involuntary admission, respondents revealed a strong support for criteria labeled danger to self, danger to others and grave disability, as grounds for admission [20]. These findings mirrored results from a previous study among psychiatrists from Illinois [21]. A Norwegian study of prototypical attitudes between groups of stakeholders towards the use of coercion, found similarly that psychiatrists tended to agree with the view that protection from harm and the patient’s need of treatment and care justify coercive measures [22]. In a study of Norwegian psychologists’ attitudes towards involuntary admission, the respondents were asked about their views on three cases of patients suffering from schizophrenia. Violence to others, followed by the patient’s obvious suffering, was here seen as the most important reasons to use coercion [23]. These findings supported the findings of a study among German and English psychologists presented with similar cases. A study of attitudes of different stakeholders from different European countries towards compulsory admission of patients with schizophrenia, found that compulsory procedures were based on traditions and personal attitudes to a considerable degree [24].

The actual process by which clinicians arrive at the decision to use involuntary admission depends in part on legal criteria. Mental health law often uses criteria such as “serious mental disorder”, “best interest”, “risk of harm” and “need of treatment and care” to regulate the use of involuntary admission. These criteria are, however, vague and leave considerable scope for discretion. The criteria may again be translated to different clinical concepts and become proxy criteria for decisions. In addition, extra-legislative factors stemming from the setting, the patient, the decision-maker, and the availability of resources are likely to influence the decision [25]. The values and beliefs of the decision-maker may then become an important determining factor.

There is a growing body of research on decision-making about involuntary psychiatric admission, yet to our knowledge, no qualitative studies of how clinicians interpret legal criteria in mental health care have been undertaken.

We wanted to investigate which choices clinicians make when asked to decide whether to admit patients that are unwilling to consent to mental health care. To illuminate the complexity of deliberation in this context, we used a qualitative research design. Specifically, we studied how experienced clinical decision-makers interpret and understand the criteria regulating involuntary admission that are laid down in the Norwegian Mental Health Care Act. The objective was to explore how different perspectives about clinician-patient interaction give rise to variations in the interpretations of criteria for involuntary admission-decisions.

## Methods

### Study design

Data was collected by means of qualitative in-depth expert interviews. Deductive thematic analysis was used: Theoretically driven questions were asked and the themes that were identified from the data were fit into the preconceived analytical frame [26]. This approach was chosen because we wanted theory-before-research to assist selecting relevant questions, to help specify what we explored during our interviews, and to aid in defining an appropriate description and interpretation of the data [27]. We aimed at providing data that may give substance to prior studies of decision making and hopefully be a vehicle for developing explanations.

### Theoretical framework

We hypothesised that different models of the clinician-patient relationship warrant different interpretations of the legal criteria for involuntary admission and different assessments of how to balance professional obligations against legal norms. We utilised Emanuel and Emanuel’s [28] ideal models of the clinician-patient relationship, specifically the paternalistic, the deliberative and the interpretive model, to highlight essential characteristics of the interaction between clinician and patient. According to the *paternalistic* model, the clinician’s goal and obligation is to ensure the best health outcome for the patient. Both the patient’s medical condition and the best intervention strategy are defined by objective criteria. Thus, the patient’s current preferences are of limited value. He will ultimately come to share the clinician’s assessment and be thankful for the decisions made on his behalf. According to the *deliberative* model, the clinician’s goal is to help the patient determine and choose values that affect or are affected by the disease and treatment. The clinician aims to persuade the patient by engaging in dialogue on the best course of action. Patient autonomy is understood as self-development relevant to medical care, and the patient’s current preferences are open to revision through moral discussion. According to the *interpretive* model, the clinician sees patient values as inchoate and often conflicting. The clinician provides the patient with information about the condition and the benefits and risks of alternative interventions, and aims at elucidating and interpreting the patient’s values through a reconstruction of the patient’s goals and commitments. The concept of patient autonomy is self-understanding, which is realised when the patient comes to know how medical interventions bear on his identity.

We chose to leave out one of Emanuel and Emanuel’s descriptions in this study (the *informative* model), because it assumes that that the patient’s preferences for intervention should be exercised without any attempt to interfere with his control over medical decision making. In the context of involuntary care, it seems prescriptively inaccurate to make use of a conception of patient autonomy that does not incorporate some capacity to reflect on and revise the understanding of the best alternative intervention (so-called second order desires).

From this background, three ideal types of attitudes-to-coercion were developed (cf. Table 1):

**Table 1:** **Comparing the attitudes- to- coercion dimensions**

	**Paternalistic**	**Deliberative**	**Interpretive**
**Patient autonomy**	Assenting to objective values	Self-development relevant to care	Self-understanding relevant to care
**Patient’s preferences**	Objective and shared	Open to revision	Requiring elucidation
**Serious mental disorder**	Lack of insight	Some insight	Some insight
**Voluntary care**	Not relevant	Should try	Should try
**Coercive care**	Best intervention	Provide care and security	Offensive intervention
**Treatability**	Effective	Uncertain effects	More harm than good
**Harm to one-self**	Individual protection	Individual care and security	Individual care and security
**Harm to others**	Individual protection	Individual protection Societal protection	Not relevant
**Overall assessment**	Balance benefits and burdens of intervention	Balance benefits and burdens of intervention, including prevention, safety, follow-up	Balance benefits and burdens of intervention, including costs of integrity loss and distrust
**Professional obligations**	Promote well-being	Through dialogue, persuade the patient of the best option	Elucidate and interpret patient values
**Professional obligations vs legal criteria**	Professional obligations trump legal criteria	Professional obligations should balance legal criteria	Critical attitude towards coercion in mental care

#### The paternalistic attitude

The patient suffering from a serious mental disorder is thought to lack insight into his own medical condition. The paternalistic oriented clinician will regard coercion as the best option available if the patient is unwilling to consider voluntary mental care. Coercive care is viewed as treatment as well as protecting the patient from harming himself or others. For the paternalist, further societal concerns are irrelevant. An overall assessment of the patient’s best interest includes considerations of benefits and harms relevant to medical care. The paternalistic clinician would follow professional obligations should these come to conflict with legal norms.

#### The deliberative attitude

The patient suffering from a serious mental disorder is thought to have some insight into his own medical condition. The deliberative oriented clinician will engage the patient in discussing the best intervention strategy and aims to persuade the patient to voluntarily accept admission. If the deliberation does not make progress, coercion might be viewed as necessary to provide care and security to the patient. Intervention protects both the patient and others from harm. An overall assessment of the patient’s best interest includes considerations of benefits and harms relevant to medical care as well as considerations of follow up-options and prevention of recurrence. The deliberative oriented clinician would try to balance professional obligations against legal norms.

#### The interpretive attitude

The patient suffering from a serious mental disorder is thought to have some insight into his own medical condition, and his values should not be judged. The interpretive practitioner views coercion as an offence. However, coercion may sometimes be deemed a necessary evil to prevent self-inflicted harm. An overall assessment of the patient’s best interest includes considerations of benefits and harms relevant to medical care including the costs regarding deprivation of liberty and the negative impact on the therapeutic alliance between the patient and the clinician, as well as the uncertainties that follow from lack of scientifically demonstrated outcome effectiveness and the prevalence of false positive predictions of dangerous acts towards others. The interpretive clinician is critical to the legitimacy of using coercive interventions in mental care.

Different declarations concerning professional ethics in psychiatry express core values of the profession. Pelto-Piri et. al. have pointed out how different perspectives are reflected in the development of medical ethics in psychiatry, and how patient rights have become more in focus in recent decades [29]. We expected the cultural shift towards less paternalism and more patient autonomy, together with health care authorities’ presumption in favor of patients’ self-determination, to be reflected in the participants’ reasoning about involuntary admission.

### Setting

Norway has one of the highest per capita incomes in the world, and is characterised by an egalitarian distribution of income and a generous social security system. Health care is need-based, universal, and tax financed. Specialised care is organised in four health regions. There is no second private tier.

Mental health care have been deinstitutionalised and decentralised in recent decades. In the period from 1990 to 2006, in-patient days declined by 43 per cent. At the same time, more patients were treated, and the expenditures in specialist mental health services had a real growth of 175 per cent from 1998–2007 [30]. In 2012, 198 involuntary admissions per 100 000 inhabitants took place [31].

The right to use coercion in mental health care is regulated by the Norwegian Mental Health Care Act of 1999. GPs refer the person to admission. A psychiatrist or hospital-based psychologists with clinical specialist qualifications make the admission decisions based on the criteria presented in Table 2. The decision-maker must reasonably believe that the person is suffering from a serious mental disorder, but it is not required a separate determination of decision-making capacity. Further, she must believe that treatment is effective or can prevent risk of harm to the person himself or to others. Involuntary admission may only be applied when voluntary care has been tried or it is obviously pointless to try this. Any involuntary observation or treatment should be in the person’s best interest and be the least restrictive alternative. Nordic mental health care legislation is based on a confidence in psychiatry as a profession and involuntary admission does not require a court verdict [22]. However, when a person is placed under compulsory mental health care, notification must be sent to a supervisory commission which is designed to be a guarantor of legal protection. This commission is appointed by the Ministry, consists of a doctor and two lay members, and is chaired by a lawyer. The patient may appeal the decision after the admission has taken place. A patient who voluntarily is under mental health care may not be transferred to involuntary care unless she constitutes a risk to herself or others.

**Table 2:** **Criteria for involuntary observation and treatment under the Norwegian Mental Health Care Act (1999 No. 62 with later amendments)**

**Criterion**	**Description**
**Disorder**	The patient is suffering from a serious mental disorder and has been examined by two independent physicians; *and*
**Voluntariness**	Voluntary care has been tried, to not avail, or it is obviously pointless to try this; *and*
**Necessary treatment**	The application of compulsory mental health care is necessary to prevent the person from having the prospects of his or her health being restored or significantly improved considerably reduced, or it is highly probable that the condition of the person concerned will significantly deteriorate in the vey near future; *or*
**Risk of harm**	The application of compulsory mental health care is necessary to prevent the person from constituting an obvious and serious risk to his or her own life and health or those of others; *and*
**Best interest**	Compulsory care may only be applied when this appears to be the best solution for the person, unless he or she constitutes an obvious and serious risk to the life or health of others.

The human rights structure already part of the Norwegian legal system was strengthened from 1999 when the Norwegian Human Rights Act stipulated that a range of international conventions, such as the European Convention of Human Rights, take precedence over Norwegian law. Thus, legal protection against involuntary admission in mental health care is based on several international resolutions and human rights conventions. The Mental Health Care Act is considered to be in accordance with the UN Mental Illness Principles [22].

### Participants

10 clinicians (psychiatrists/psychologist) with extensive experience with admission decisions, working at three different health enterprises from different parts of Norway, were interviewed in 2013. The sample was partly identified ex ante according to the position and experience the participants held, and partly by chain-referral, where the initial set of relevant participants was supplied with clinicians nominated by those already interviewed. Thus, we used a non-probability sampling approach: The participants were chosen exactly because they were regarded as experienced and well-knowing decision-makers [32].

The number of participants is small. Had we used an inductive approach, such as grounded theory, we would have included participants until no novel topics were raised during the interviews (saturation). In this study, however, we did not aim to explore a range of themes grounded in the data itself. An analytical framework was used to aid in defining an appropriate description and interpretation. We wanted to find out how the participants framed their views, why they held these views and how they made connections among options. The number of participants was determined to be adequate to answer our research questions, given limited time and resources.

### Interviews

Data was collected by means of qualitative in-depth expert interviews. The interviews were semi-structured and lasted 1- to 1,5 hours. They were based on an interview guide which was thematically organised. The participants were asked about their reflections about: Variation in involuntary admission rates; the legal criteria for involuntary admission; the communication with patients about voluntary and involuntary admission; the availability of resources; and the reasons behind discharge decisions. Open-ended responses were allowed to uncover as much information as possible. The participants were asked to ensure that they did not reveal the identity of any patients when they discussed challenging situations.

All interviews were conducted in Norwegian and audio-taped. The most relevant parts were transcribed and translated into English. Each participant was given the opportunity to review the relevant transcript. The transcribed data was then grouped into two themes, which again were divided into sub-issues. The first theme, reflections on the legal criteria for involuntary admission, was divided into sub-issues according to different legal criteria (*mental disorder; voluntariness; necessary treatment; risk of harm to oneself; risk of harm to others; overall best interest*). The second theme, reflections on professional role and integrity, was divided according to *institutional factors* and *external pressure*.

### Ethical approval

Since the purpose of this study was to generate knowledge about health services (and not health per see), and data gathered and stored was information about clinicians’ attitudes and beliefs, the study was not covered by the Norwegian Health Research Act (and should not be submitted to the Regional Committee for Medical and Health Research Ethics). The study was submitted to the Data Protection Official for Research, Norwegian Social Science Data Services. It was approved in relation to the Norwegian Data Act (project no.33762).

## Results

### Understanding and interpreting the legal criteria

#### Mental disorder

A necessary condition for involuntary mental health care to be applied is that the patient is suffering from a serious mental disorder. This term is a legal one and not a medical diagnosis. The Norwegian Supreme Court has interpreted the term as to include a psychosis or as a reduction in functioning equivalent to what is seen in psychosis [33]. The participants in our study had a similar understanding:

*“A serious mental disorder is defined and described as a psychosis (…). A psychosis is a psychosis and in reality you don’t need a specific diagnosis (…). Then you have the other group, well, the thought is that you will identify a group with very severe personality disorders. The functional failure is described as comparable to a psychosis”.*

*“A serious mental disorder is a description of a person’s functioning more than a diagnosis, it is about a person’s ability to understand reality and her understanding and ability to make choices regarding her own mental condition”.*

#### Voluntariness

According to the Mental Health Care Act, voluntary mental health care must have been tried, to not avail, or it has been found obviously pointless to try this before involuntary mental health care is applied. Most of the participants did not consider this criterion to be useful in their decision-making. Some reported that promoting voluntary options were not prioritised. Although they thought that a deliberation with the person to find a solution together would be the best decision making procedure, it was rarely tried out. The common attitude seemed to be that trying voluntary solutions were pointless in most cases:

*“If the patient is considered to be suffering from a serious mental disorder, the patient is obviously not autonomous and thus not able to make rational decisions. So why should I try and convince the patient to voluntarily receive treatment?”*

*“You will very soon get an impression of whether the person will volunteer or not”.*

However, the participants also made the point that the Norwegian prohibition against transfer from voluntary to involuntary mental health care adds to the complexity when interpreting the voluntariness-criterion. One participant stated that clinicians may not put much effort into deliberation about voluntary care, because of the high risk for involuntary readmission after discharge. From the clinician’s point of view, this procedure seemed *“inconvenient”* and “*ineffective*”. Another of the participants pointed out that the voluntariness- criterion seems to be grounded in a poor understanding of the actual choice situation of the decision-maker:

*“When I hear politicians and others in media say that voluntary solutions are always best (…) I think that: You have no idea what you are talking about”.*

#### Necessary treatment

Most decisions on involuntary mental health care in Norway are based on expectations about effect of treatment [34]. The treatment-criterion is described as having the prospect of health being restored or significantly improved considerably reduced, or it is highly probable that the condition will significantly deteriorate in the very near future. Some of the participants reported that they often based involuntary admission on this criterion. The assessment of effect seemed to be grounded either on information about “*the previous experience with the actual patient*” or on “*a comparison with similar cases*”. Others, however, found this criterion difficult to understand and to use. Those who regarded this criterion as “*difficult*” had different reasons for their evaluation. One of the participants believed that withholding treatment *“almost never”* resulted in considerable worsening of condition, and pointed out that “*the coercive aspect of the admission and treatment might deteriorate the possibility of effect*”. Others draw attention the fundamental difficulty of evaluating whether or not the patient would experience considerable improvement from treatment. In some cases, treatment would be expected not to have effect:

*“These are patients that have symptoms of psychosis even when they are medicated and when committed, they get restless and aggressive, making their condition worse”.*

The treatment-criterion seemed, on the other hand, to be used more frequently in decisions about discharge.

#### Risk of harm to oneself

If mental health care is necessary to prevent a person constituting an obvious and serious risk to his or her own life and health or that of others, involuntary admission may be applied. The harm-to-oneself criterion was regarded as met by all the participants if the person involved was suicidal. The current legislation does not, however, indicate that risk of suicide is a sufficient condition for involuntary mental care, and this was pointed out by most participants. Still, several of the participants reported that the health enterprises were recommended to apply involuntary mental health care to suicidal persons even when the disorder-criterion is not met, and found this practice to contradict the intention of the law:

*“The hospital emergency centers and the regional psychiatric centers have received strong signals that when it comes to suicidal patients, they should always use compulsory observation (…) It is several years since the mental health care have practiced this in a proper legal manner”.*

*“When it is claimed by a patient that he is suicidal, most health care practitioners are not that concerned about whether or not the main criterion of serious mental disorder is present (…). I think this is wrong. It is actually legal to commit suicide”.*

The political signals from health care authorities on this issue were regarded as confusing:

*“The government wants it both ways. They (the government) want the use of force to be reduced, but at the same time they want to make sure that no one takes his own life”.*

#### Risk of harm to others

To determine whether or not the harm-to-others criterion should be applied, the previous experience with the person was considered relevant. The participants found this criterion difficult to use, however, both because the risk of harm to others was found to be “*inherently difficult to predict*” and because this criterion is not sufficiently specified. As one of the participants said:

*“The harm-to-others-criterion is (…) a pretty challenging text to interpret. The harm-to-others-criterion does not set a time limit in the same way as the treatment-criterion”.*

#### Best interest

According to the legal text, an overall assessment of how beneficial involuntary mental health care may be for the person concerned must be carried out, even though the other criteria are met. The participants reported that they used a range of different criteria when assessing the overall benefit of involuntary care, such as: *Need; Degree of opposition against care; Expected benefit of care; Expected functioning outside institution; Professional follow-up; Housing, social network, and dependents; Previous experience with the person*.

The best interest criterion was understood as an informal decision rule by the participants. They did not use a checklist or an assessment tool. Some considered a variety of different factors, others only a few. Expected functioning outside institution was considered by most. The participants would evaluate the social and family network of the person, and the person’s ability to eat, sleep and control her impulses outside institution. Some thought that the present housing situation and professional follow-up routines were very important:

*“I would look into the practicalities (…) Are the services the patient needs available? Does the patient have a place to live?”*

*“If a sufficient number of (…) adequate apartments and services are provided, then I would imagine the use of compulsory admissions to be reduced”.*

Considerations about the present family situation were similarly pointed out:

*“If for instance there are children in the home, one would maybe to a larger extent continue treatment than one would if there are no children in the home”.*

### Understanding the professional role and integrity

#### Institutional factors

All of the participants reported that institutional factors, such as the culture of the institution in which they worked and the professional norms developed through practice, would be relevant for their decision making. One said:

*“The colleagues will influence your decisions. You are socialised into a certain way of thinking”*.

Institutional factors also included the practices of the supervisory commission. Several of the participants pointed out that over time, the use of involuntary mental care will be ”*shaped by the practice of the supervisory commission*”.

When asked if one thought colleagues at other institutions would interpret the legal text differently from themselves, the answers varied. Some of the participants based their impression about colleague’s decision making on discussions within the profession and claimed that there was a “*mutual understanding of the use of involuntary admission*”. Others, however, stated that many clinicians did not know the content of the law:

*“Many do not even know that suicidal disposition should be assessed against the additional criteria, but believe that this alone is enough to commit someone”.*

#### External pressure

In Norway, there have been several political initiatives to reduce the use of coercion in mental health care. The participants reported that they did not take political recommendations into consideration when making decisions about involuntary admissions:

*“I will not be influenced by signals from for instance the Minister of Health. I would consider him as an authority, yes, but he does not have thirty years of experience. He is a person that has received information from others. Maybe inexperienced physicians would listen”.*

*“Physicians and other health care practitioners making these decisions are usually very independent and confident in their own evaluation, and will not let non-professionals influence their decisions”.*

## Discussion

The criteria listed in the Norwegian Mental Health Care Act specify the circumstances in which a person may be placed under involuntary mental health care. The Act applies what has been called a *status approach* [8], whereby the presence and severity of a mental disorder and the need for treatment or perceived risk to the patient or others is necessary for the legality of use of coercion. The disorder-criterion is briefly stated in the legal text. It does not use medical diagnoses, nor is a list of disorders of mental function given. Further, *a clinical model* of substitute decision-making [35] is prescribed. The clinician is appointed to act in the best interest of the person if the person lacks capacity to understand the nature and purpose of treatment options and to make informed and reasoned decisions about treatment.

As pointed out by Dawson, criteria such as these are “considered, applied, stretched, and occasionally ignored in key decisions in compulsory assessment and treatment procedures” [12]. In the following, we discuss the participants’ interpretation of the criteria.

The participants knew the content of relevant criteria and they accepted the authority of the legal text. However, the study shows how clinical decision-makers are given wide latitude to act in the health interests of the mentally disordered. The participants seemed to make use of a functional interpretation of the disorder-criterion. There was a tendency to equate serious mental disorder with loss of decision-making capacity. The abilities needed for persons to decide for themselves about treatment seemed to receive little attention. Although a deliberation about different options with the person was described as an ideal decision-making procedure, using time to convince the person of mental care was regarded as pointless in most cases. The person’s capacity for autonomous choice was not formally assessed. Rather, the participants described how they would assess decision-making capacity discretionally. This finding was categorised as an indication of paternalistic oriented reasoning. According to the paternalistic attitude-to-coercion, a person lacking capacity should be given treatment against his will if there is reason to believe that is in the person’s best interest and the option to be preferred by him had he been of sound mind.

Norwegian legal standards for involuntary admission are defined in terms of mental disorder linked to consequences if not treated or cared for. The references to treatment given in the Act are controversial, as a prognosis is difficult without a diagnosis. We found different opinions regarding the treatment-criterion represented among the participants, indicating that paternalistic, deliberative and interpretative oriented arguments were used. While some of the participants found this criterion useful, and used information from prior treatment of the person or similar cases to assess the likely effects of treatment, others pointed out the difficulties of using this criterion in situations where no effective treatment is available, or where the negative effects of involuntary treatment are perceived to outweigh the positive treatment-effects. The participants’ statements about the treatment-criterion seemed to reflect the ongoing scientific debate about effect of involuntary treatment of mental disorders, given the lack of reliable effect-studies [34].

The risk-of-harm-criterion poses specific challenges, as it is inherently difficult to predict rare events such as suicide or violence. Studies have shown that that risk of suicide is insufficient warrant for involuntary treatment [36], and there is no evidence that violence is more predictable in those with mental disorders [37]. The participants in our study regarded the condition of risk of harm to oneself to be met if the person involved was suffering from a mental disorder and was suicidal. In these situations, involuntary admission would be used. However, the practice of protecting persons without a mental disorder from taking their own life was interpreted as to be against the intention of the law. Further, the risk-of- harm-to-others seemed irrelevant to the participants. Their main focus was the health of the individual person, not public safety. These statements were interpreted as representing paternalistic oriented reasoning about the health interests of the mentally disordered versus the protection of others.

The Act has incorporated a best-interest-criterion whereby the clinical decision-maker is required to act in the best interest of the person. No formal assessment tool is developed. We found that assessment of best interest carries a large burden in the decision-making process. The participants reported a range of different conditions that were taken into consideration. The best-interest-criterion opens for potentially large variations in practice. Both socialisation processes involving colleagues and the respective supervisory commissions will come to play important roles in shaping practice. The participants seemed to give much weight to expected functioning outside institution but did not mention considerations of factors like integrity loss or distrust, as we would have expected if interpretive arguments were used.

### Deliberative ideals – paternalistic practices?

Since the beliefs about patient autonomy and decision-making capacity have shifted and a less paternalistic approach to the clinician-patient relationship has developed, we expected the participants in this study to base their reflections about involuntary admission on deliberative- or interpretive-oriented arguments.

The investigation revealed, however, that much of the participants’ reasoning fit into a paternalistic framework. The participants used their skills to observe and determine the patient’s medical condition and to identify whether he was suffering from a serious mental disorder. Severely disordered patients were assumed to have low level of insight, and in most cases it was considered pointless to try voluntary mental health care. Involuntary admission was assessed necessary for attaining autonomy and dignity. Further, the participants pragmatically choose to adhere to local health authorities’ recommendation to admit suicidal patients even if the necessary legal criteria for involuntary admission were not met. The participants were reluctant to assess whether or not the patient was at risk of harming others, and hesitated to assess societal consequences of untreated mental disorder.

At the same time, deliberative oriented reasoning was found. Some of the participants explained that they preferably would follow a deliberative ideal regarding patient autonomy and decision-making capacity. Some regarded involuntary admission as necessary to provide care, but considered the treatment effect of coercion as uncertain. Some considered a range of diverse factors to be of importance when assessing whether involuntary admission was the overall best solution for the patient involved. This included factors external to the admission-decision itself, such as housing, social network, and follow up-options.

Our study suggests that a deliberative ideal lapses into paternalistic oriented reasoning in practice. The participant attitudes may seem puzzling. Self-contradictory findings in this field have, however, been reported previously. A study of attitudes towards paternalism, patient autonomy and moral deliberation in the clinician-patient relationship, found that 37 per cent of the whole population of doctors was ambivalent [38]. In a study of staff members encounters with patients in psychiatric care, a majority of the statements could be attributed to a paternalistic perspective, but there was also an awareness of patients’ right to autonomy [29]. Another study concluded that psychiatrists tended to agree with paternalistic attitudes towards the use of coercion in mental health care, but still disagreed with the statement that effective treatment is more important than patient self-determination [22].

Several factors may explain why paternalistic oriented reasoning prevails. Insecurity in the professional role may push practitioners towards a paternalistic attitude in order to reduce medical uncertainty [38]. However, the participants in this study revealed strong professional identity and trust in their professional role. Another factor that may be involved is the sense of urgency that describes the choice situation. We may speculate that participants in this study view the decision-making in this context as an emergency given the limit of resources and time. According to Emanuel and Emanuel’s discussion of ideal interaction, a paternalistic response would then be justifiable:

“Clearly, under different clinical circumstances different models may be appropriate (…) Thus it is widely agreed that in an emergency where delays in treatment to obtain informed consent might irreversibly harm the patient, the paternalistic model correctly guides physician-patient interaction” [28].

As pointed out by Pelto-Piri et. al., shifting from paternalism to a perspective where the focus is on the interaction between the professionals, the patient and her family may involve greater complexity and seems difficult in practice [29]. Yet, the intention of the Norwegian Mental Health Care Act is violated if actual decision making behavior accord with a paternalistic attitude. Of course the Act cannot address all the complex issues raised in this context, and the legal criteria cannot replace moral deliberation among clinicians. The doctrine of proportionality requires, however, that any exemption from the European Human Rights Convention must be to the minimum extent possible. If the findings bears a resemblance to current thinking among the clinician population, a greater awareness of how human rights are incorporated in the legislative background against which the Mental Health Care Act is developed, is called for.

### Limitations

Four important limitations are worth pausing over. First, we used a deductive theoretical approach and developed three attitudes-to-cohesion ideal types to guide our analysis by focusing on crucial aspects of different types of interaction, free from complicating details. Decision-making is complex in mental health care. When applying preconceived categories, important factors may have been left out of the study. Second, the aim of this study was not to generalise from this sample to the population, but to obtain information about important aspects of decision making with regard to involuntary admission from those who are closely involved (i.e. clinical decision-makers). The sample is small, and we do not know to which extent the sample resembles the population of interest. Third, we do not know how the participants actually make decisions. Their attitudes may not accord with actual behavior. Fourth, expert interviews may of course be framed by partial responses. The participants were well-educated and highly knowledgeable subjects. They may have wanted to slant their accounts in order to appear consistent, rational and independent of pressure. These limitations are inherent in the design of this study.

## Conclusions

Legal criteria for involuntary admission under the Norwegian Mental Health Care Act are open for discretion by the decision-making clinician. Considerably variation in how the law is understood, interpreted and operationalised may emerge. This study has shown how reasoning about legal criteria is influenced by clinicians’ understanding of paternalism, patient autonomy and moral deliberation. We expected the recent shift towards less paternalism within health care to be mirrored in this study.

Findings suggest, however, that a deliberative ideal of the clinician-patient relationship and corresponding attitudes-to-coercion lapses into paternalistic oriented argumentation in practice. The participants regarded considerations of the vulnerability of the person to be more important than considerations about lack of capacity to make decisions about their own health risks. Further, the costs of treatment imposed on an unwilling person relative to benefits were minimised. The participants were reluctant to give much weight to predictive risk assessments concerning public safety. Decisions about involuntary admissions also seem to be informed by a range of extra-legislative factors, such as the patient’s need, degree of opposition against care, expected benefit of care, expected functioning outside institution, professional follow-up routines, housing, network and dependents, and previous experiences with the patient. These findings indicate the complexity of moral deliberation regarding these decisions. The role of different values in balancing favorable effects and disadvantages of the use of coercive measures must be recognised. This balancing is a matter of clinical professionals’ discretion. Thus, legal norms cannot be the only resource guide for decision-makers, and should be supplemented with professional guidelines.

Modern psychiatry has been described by Welsh and Deahl as “uncomfortably wedged between the territories of law and medicine, between coercion and care” [3]. They pointed out that identification of the reasons why psychiatrists make certain decisions, and assessment of these choices for consistency and consequences, is important for the profession to begin to develop moral justification. This study adds data which hopefully will advance research in this field.
